# Additive-Enhanced Exfoliation for High-Yield 2D Materials Production

**DOI:** 10.3390/nano11030601

**Published:** 2021-02-28

**Authors:** Dinh-Tuan Nguyen, Hsiang-An Ting, Yen-Hsun Su, Mario Hofmann, Ya-Ping Hsieh

**Affiliations:** 1 Department of Materials Science and Engineering, National Cheng Kung University, Tainan 70101, Taiwan; tuanngdvn@gmail.com (D.-T.N.); yhsu@mail.ncku.edu.tw (Y.-H.S.); 2 Department of Mechanical Engineering, National Chiao Tung University, Hsinchu 30010, Taiwan; andyting86@gmail.com; 3 Department of Physics, National Taiwan University, Taipei 10617, Taiwan; 4 Institute of Atomic and Molecular Sciences, Academia Sinica, Taipei 10617, Taiwan

**Keywords:** TMD, WS_2_, additive-assisted exfoliation, 2D materials

## Abstract

The success of van-der-Waals electronics, which combine large-scale-deposition capabilities with high device performance, relies on the efficient production of suitable 2D materials. Shear exfoliation of 2D materials’ flakes from bulk sources can generate 2D materials with low amounts of defects, but the production yield has been limited below industry requirements. Here, we introduce additive-assisted exfoliation (AAE) as an approach to significantly increase the efficiency of shear exfoliation and produce an exfoliation yield of 30%. By introducing micrometer-sized particles that do not exfoliate, the gap between rotor and stator was dynamically reduced to increase the achievable shear rate. This enhancement was applied to WS_2_ and MoS_2_ production, which represent two of the most promising 2D transition-metal dichalcogenides. Spectroscopic characterization and cascade centrifugation reveal a consistent and significant increase in 2D material concentrations across all thickness ranges. Thus, the produced WS_2_ films exhibit high thickness uniformity in the nanometer-scale and can open up new routes for 2D materials production towards future applications.

## 1. Introduction

The integration of two-dimensional materials into van-der-Waals thin-film electronics has the potential to enable future electronics with unprecedented possibilities [1,2,3,4]. The compatibility of 2D materials with solution processing provides a route to print electronics at large scale directly [5,6,7]. Additionally, the readily available and earth-abundant bulk precursors for transition metal dichalcogenide-based 2D materials, such as WS2 and MoS2, permit their cost-effective and ecological production compared to current materials [6]. Secondly, the intrinsic atomic thickness of 2D materials imparts electronic devices with previously unachievable flexibility and transparency. Combining these attractive commercial and fundamental properties could enable emergent wearable, implantable, and textile-like devices in ubiquitous sensors and interfaces [7].

Shear exfoliation has emerged as one of the most promising production techniques for this task due to the high achievable quality, scalability, and energy efficiency of production [8,9,10]. However, to date, the method’s yield rate is still very modest, less than 0.1 mg·ml^−1^ h^−1^ [11], which is two to three orders of magnitude smaller than required for industrial-scale production [12]. Improving the process is particularly challenging for TMDs as the shear rate needed to exfoliate few-layer TMDs is three times higher than that of graphene [13].

To satisfy the demands from its applications, significant effort has been invested into increasing the exfoliation yield by optimizing the shear exfoliation process [11,14,15,16,17]. Unfortunately, there are several limitations and competing processes that provide boundaries to the optimization. The mechanical design of the shear mixer employed for exfoliation, for example, sets restrictions to the exfoliator dimension and achievable rotor speed. Moreover, the increase in rotor-speed results in lower materials quality, the increase in precursor concentration exacerbates the re-stacking of nanoflakes into thicker ones [8], and extension of exfoliation durations decreases power efficiency. Thus, new approaches towards increasing the exfoliation yield while retaining the other beneficial aspects of liquid exfoliation have to be devised.

**Figure 1:**
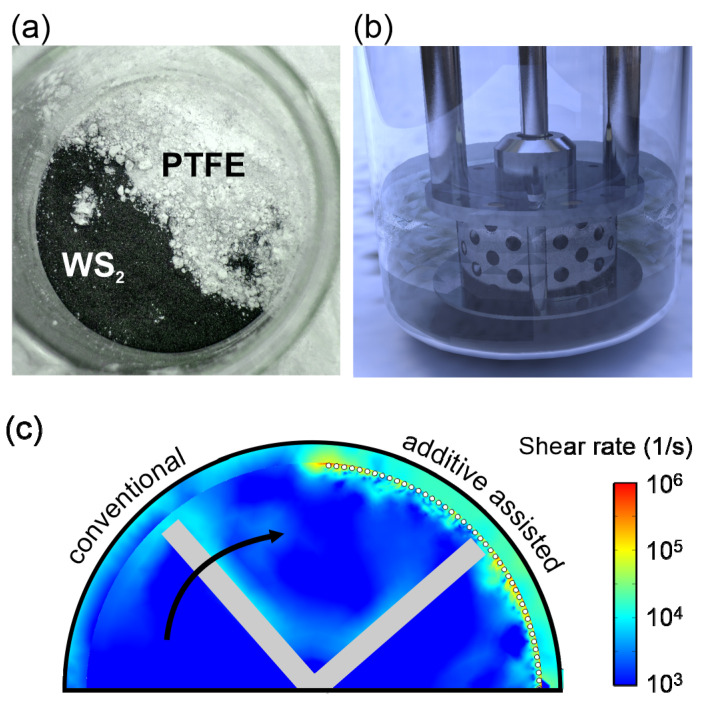
Schematic of additive-assisted exfoliation, (**a**) photograph of WS_2_ powder and PTFE additive, (**b**) schematic of the experimental setup, (**c**) Simulation of shear rate enhancement due to mechanical rotor-stator gap decrease.

We pursue an approach to dynamically modifying the shear rate in the rotor-stator-gap. Previous work established the importance of the gap dimension between a shear exfoliator’s stator and rotor towards the exfoliation yield [8,18]. Despite the importance of this parameter, little effort has been made to minimizing the rotor-stator-gap since it is usually a fixed parameter of a given shear exfoliator design [18]. Here, we alter the gap by adding a material that does not itself become exfoliated and whose properties do not change the exfoliation mechanics (Figure 1a–b). Instead, the inert additive enhances the shear rate by mechanically modifying the gap dimension. Finite element, fluid dynamic simulation of a shear mixer demonstrates a significant increase in shear rate if particles are added in the rotor-stator gap (Figure 1c) (more information is available in Figure S1 in the Supplementary Material).

We demonstrate the increased exfoliation efficiency of this additive-assisted exfoliation (AAE) process on the example 2D transition metal dichalcogenides such as MoS_2_ and WS_2_ as these have demonstrated attractive properties for van-der-Waals electronics, such as high carrier mobility [19] and strong optoelectronic responsivity [20]. We observe an enhancement of exfoliation yield through AAE by up to 480% compared to conventional exfoliation. Spectroscopic and microscopic evaluation indicates the high quality and good uniformity of the exfoliated material. Our generally applicable approach enhances the potential of 2D materials-based van-der-Waals thin films for future electronics. 

## 2. Materials and Methods

### 2.1. Exfoliation

In a typical experiment, 9 g of WS_2_ (2 µm, 99% purity, Sigma-Aldrich, Darmstadt, Germany) is mixed with 6 g of polytetrafluoroethylene (PTFE) particles (Fluorez Technology, Taiwan, 1–7 µm) and then added with 250 mL n-methyl-2-pyrrolidone (NMP) solvent (99.5% HPLC grade, Sigma-Aldrich). The solution is shear mixed in a homogenizer (Silverson LM-5A, Silverson, MA, USA) at 8000 rpm rotor speed for 90 min. The bottle containing the solution is submerged in an ice-filled tank connected to a chiller that keeps a constant temperature of 8 °C.

### 2.2. Size Selection

The supernatant of the exfoliated solution is decanted into 50 mL centrifuge tubes and centrifuged for 1 h at 1200 rpm. The sediment from this stage containing unexfoliated flakes is discarded, while the supernatant is centrifuged further at 2000 rpm. After that, the sediment is re-dispersed with 50 mL NMP, while the supernatant is centrifuged further at 7500 rpm. The collected solutions (re-dispersed sediments and supernatants) are then filtered through glass microfiber filter papers (0.45 µm pore size, Whatman, UK).

## 3. Results and Discussions

The proposed AAE process relies on the judicious choice of an additive. Unlike previous approaches, such as shear grinding exfoliation [21] and zeolite-assisted exfoliation [22], the envisioned additive decreases the rotor-stator gap without modifying the exfoliation process or getting consumed during exfoliation. Therefore, suitable additives must be chemically stable in the exfoliation solution and be mechanically tough enough not to fragment in shear exfoliation but not too hard to cause damage to the mixer’s head. Finally, the ease of removing the additive from the exfoliated material is another factor to consider. After surveying a number of additives, including nickel, diatomaceous earth, zeolite powder [22] and paint-coated silica particles for these requirements (Table S1 in the Supplementary Material), we identified polytetrafluoroethylene (PTFE) spheres of 4 µm diameter as the most promising candidate. PTFE has a low hardness of 4.8 [23], is chemically inert in commonly employed solvents and does not exfoliate.

**Figure 2:**
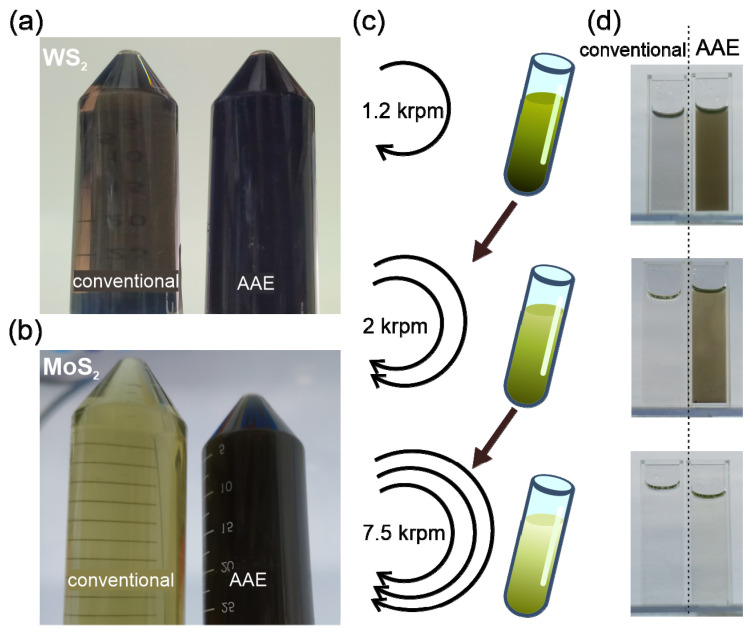
Optical characterization of AAE results: (**a**) photograph of conventional exfoliation and AAE solutions after exfoliation, (**b**) UV-visible absorption spectra of AAE and conventional solutions after 1.2 krpm centrifugation with indication of A-exciton peak at 640 nm, (**c**) schematic of liquid cascade centrifugation stages and (**d**) corresponding photographs of AAE and conventionally exfoliated solutions.

To demonstrate the potential of AAE, we conducted shear exfoliation following previous reports [13]. The enhanced exfoliation efficiency of AAE can be directly inferred from simple optical inspection. After exfoliation, the AAE solution is darker than the reference solution, suggesting a higher concentration of WS_2_ (Figure 2a). This observation agrees with weight measurements, which show that the amount of WS_2_ nanoflakes produced by AAE (2.8 ± 0.4 g) more than doubles that produced by the reference process (1.2 ± 0.1 g) (details in Supplementary Material). The literature review demonstrates that the AAE yield represents one of the highest values reported for WS_2_ exfoliation, which can be further enhanced by optimizing the process parameters and emphasizes the importance of our advance (Table 1). Moreover, the AAE mechanism’s universal applicability to other 2D materials is demonstrated by the enhanced contrast in AAE-exfoliated MoS_2_. (Figure 2b) (details in Supplementary Materials).

While the observed difference in optical contrast indicates a larger concentration of 2D material after exfoliation, such a simple analysis is not sufficient to determine the production yield in the presence of differences in the solution and additive concentrations. To identify the impact of AAE on the production yield of thin WS_2_ flakes, we conducted liquid cascade centrifugation (LCC) to separate flakes with different dimensions according to their relative buoyancy [13] (Figure 2c). We observe that the contrast of AAE WS_2_ is higher for each centrifugation stage (Figure 2d), suggesting that the higher exfoliation yield does not affect the distribution of flake sizes.

**Figure 3:**
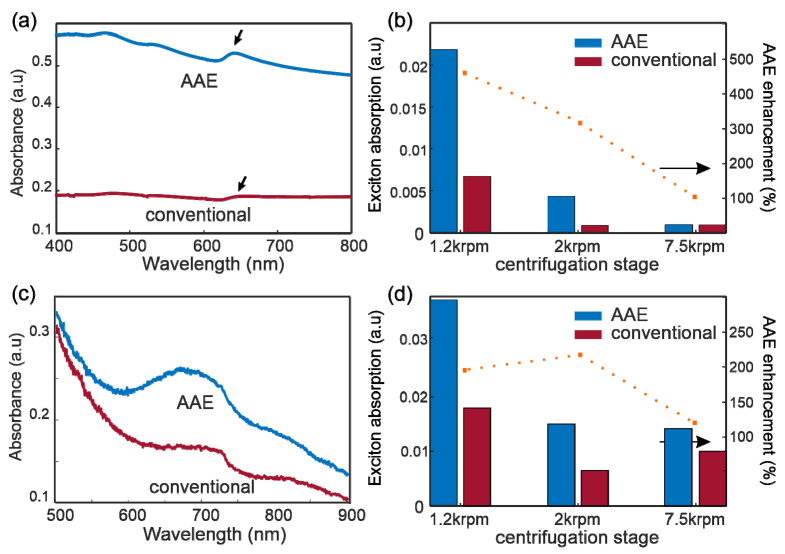
Spectroscopic evidence of AAE enhancement: (**a**), UV-vis spectra of exfoliated WS_2_ solutions produced from AAE and conventional shear exfoliation, with arrows indicating A-exciton peak (**b**) exciton absorption of WS_2_ samples produced from AAE and conventional shear exfoliation (columns) and AAE improvement (dotted line), (**c**) UV-Vis spectra of MoS_2_ solution for AAE and conventional exfoliation, (**d**) exciton absorption of MoS2 and AAE improvement.

The AAE enhancement was quantified by UV-visible absorption spectroscopy of WS_2_ and MoS_2_ solutions after exfoliation and LLC. The obtained spectra for both WS2 and MoS_2_ demonstrate peaks in the solvent background that agree with previously reported exciton features [24,28,29] (Figure 3a,c). The intensity of the A-Exciton is significantly increased for AAE compared to conventional exfoliation, corroborating the enhanced yield. An algorithm using asymmetric polynomial smoothing [30,31] is employed to remove the response of PTFE additive and calculate the exciton intensity. We investigate the variation of exciton intensity with centrifugation cascade and observe a large and consistent increase in concentration for all ranges of flake thicknesses. The enhancement of AAE over conventional WS_2_ exfoliation increases from 320% for 1.2 krpm to 480% for 2 krpm, indicating its advantages for producing thin flakes (Figure 3b). The low concentration of thinner flakes makes the spectral characterization unsuitable for assessing the enhancement at a high centrifugation speed.

The enhancement of AAE can also be observed for MoS_2_ exfoliation (Figure 3d), but the lower value of enhancement raises questions about its limitations. The underlying mechanism of AAE is the increase of exfoliation power by the decrease of the rotor-stator gap [18]. This increased kinetic energy, however, generates both exfoliation and fragmentation. The increase of exfoliation yield with increasing power is thus offset by the decrease in flake dimension through shattering. Consequently, the yield of sufficiently large and thin flakes depends on the ratio of intralayer bonding strength to interlayer bonding strength. AAE enhancement is expected to be larger for materials with high interlayer bonding strength and weak intralayer bonding strength, such as graphite and WS_2_, but reduces for weaker materials with stronger interlayer bonding, such as MoS_2_ and hexagonal boron nitride (hBN) [11,13,32].

**Figure 4:**
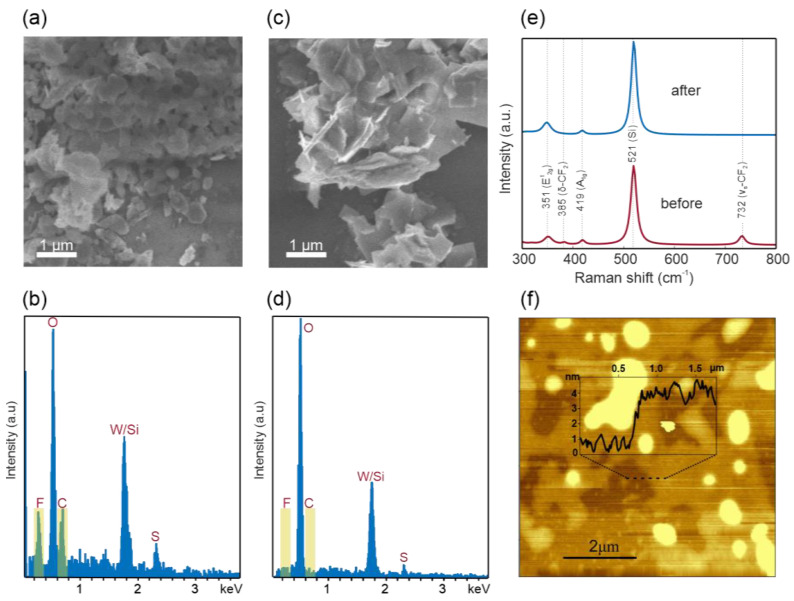
SEM image (**a**) and EDX spectra (**b**) of WS_2_ sample before filtering; SEM image (**c**) and EDX spectra (**d**) of WS_2_ sample before filtering; (**e**) Raman spectra of WS_2_ samples before and after filtering (**f**) Atomic force micrograph of WS_2_ flakes obtained by AAE.

To enable applications in van-der-Waals electronics, AAE has to be able to produce material with high quality. As a first step towards this goal, we demonstrate that the additives can be easily removed after AAE. Scanning electron microscopy and energy-dispersive X-ray analysis of AAE material deposited reveal large clusters that are assigned to fluorocarbons, which confirms the presence of PTFE after exfoliation (Figure 4a,b). A simple filtering step using Whatman glass fiber membrane (0.45 micron) results in the suppression of such clusters, and no fluorocarbon can be detected (Figure 4c,d). To corroborate these findings, Raman spectroscopy was carried out on the samples before and after filtering (Figure 4e). In addition to the characteristic features of WS2 (E^1^_2g_ peak at 351 cm^−1^ and A_1g_ peak at 419 cm^−1^) [33] and Si (520 cm^−1^), the Raman spectrum of exfoliated WS_2_ sample also displays fingerprints of PTFE (peaks at 732 cm^−1^ and 385 cm^−1^) [34,35]. After filtering, the peaks associated with PTFE disappear, demonstrating the effectiveness of additive removal.

After centrifugation and filtering, ultrathin 2D material flakes with high thickness uniformity can be obtained from AAE. Atomic force micrographs of Langmuir–Blodgett-type films [36] demonstrate that uniform films of ~3 nm thickness can be produced (Figure 4f), which, according to a previous report [37], corresponds to an average nanosheets’ thickness of ~4 layers (more characterization results are provided in Figures S2 and S3 in the Supplementary Materials). 

## 4. Conclusions

In conclusion, our work presents a novel method to improve the concentration of 2D materials produced by the shear exfoliation of transitional metal dichalcogenides. The introduction of PTFE particles as additives increases the exfoliation efficiency of thin 2D material flakes by more than 400% and achieving 30% exfoliation yield for few-layer nanoflakes. After exfoliation, the additives can be removed completely by simple vacuum filtering, yielding uniform and nanometer-thick van-der-Waals thin films. Our results open up new routes towards liquid-based electronics.

## Figures and Tables

**Table 1:** Comparison of WS_2_ yield and thickness for different exfoliation methods.

Solvent	Method	Duration	Thickness	Yield	Reference
SC/H_2_O	Li-preintercalated sonication	2 h sonication (+3 days intercalation)	2–3.6 nm	18–22%	Xu et al. (2018) [24]
NH_3_/H_2_O	Tip sonication	3 h	2–8 nm	25%	Adilbekova et al. (2020) [25]
NMP	Ball milling + sonication	12 h milling + 1 h sonication	3–5 nm	20.1%	Han et al. (2017) [26]
DMSO/H_2_O	Microwave-assisted sonication	2 h	3–12 nm	18%	Ma et al. (2020) [27]
NMP	Additive assisted shear exfoliation	1.5 h	3–8 nm	31%	This report

## Supplementary Materials

The following are available online at https://www.mdpi.com/2079-4991/11/3/601/s1, Figure S1: Calculated shear rates of AAE and conventional exfoliation, Figure S2: WS2 flake size distribution, Figure S3: WS2 thickness distribution. Table S1: Comparison of various additives.

## References

[1] Lin Z., Huang Y., Duan X. (2019). Van der Waals thin-film electronics. Nat. Electron..

[2] Zhang Y., Zheng B., Zhu C.F., Zhang X., Tan C.L., Li H., Chen B., Yang J., Chen J.Z., Huang Y. (2015). Single-layer transition metal dichalcogenide nanosheet-based nanosensors for rapid, sensitive, and multiplexed detection of DNA. Adv. Mater..

[3] Seo J.-W.T., Zhu J., Sangwan V.K., Secor E.B., Wallace S.G., Hersam M.C. (2019). Fully inkjet-printed, mechanically flexible MoS2 nanosheet photodetectors. ACS App. Mater. Inter..

[4] Ramasubramaniam A., Naveh D., Towe E. (2011). Tunable band gaps in bilayer transition-metal dichalcogenides. PhRvB.

[5] Lin Z.Y., Liu Y., Halim U., Ding M.N., Liu Y.Y., Wang Y.L., Jia C.C., Chen P., Duan X.D., Wang C. (2018). Solution-processable 2D semiconductors for high-performance large-area electronics. Nature.

[6] Chhowalla M., Shin H.S., Eda G., Li L.-J., Loh K.P., Zhang H. (2013). The chemistry of two-dimensional layered transition metal dichalcogenide nanosheets. Nat. Chem..

[7] Khan S., Lorenzelli L., Dahiya R.S. (2015). Technologies for Printing Sensors and Electronics Over Large Flexible Substrates: A Review. IEEE Sens. J..

[8] Paton K.R., Varrla E., Backes C., Smith R.J., Khan U., O’Neill A., Boland C., Lotya M., Istrate O.M., King P. (2014). Scalable production of large quantities of defect-free few-layer graphene by shear exfoliation in liquids. Nat. Mater..

[9] Large M.J., Ogilvie S.P., Amorim Graf A., Lynch P.J., O’Mara M.A., Waters T., Jurewicz I., Salvage J.P., Dalton A.B. (2020). Large-Scale Surfactant Exfoliation of Graphene and Conductivity-Optimized Graphite Enabling Wireless Connectivity. Adv. Mater. Technol..

[10] Liu L., Shen Z., Yi M., Zhang X., Ma S. (2014). A green, rapid and size-controlled production of high-quality graphene sheets by hydrodynamic forces. RSC Adv..

[11] Varrla E., Backes C., Paton K.R., Harvey A., Gholamvand Z., McCauley J., Coleman J.N. (2015). Large-Scale Production of Size-Controlled MoS2 Nanosheets by Shear Exfoliation. Chem. Mater..

[12] Yi M., Shen Z. (2015). A review on mechanical exfoliation for the scalable production of graphene. J. Mater. Chem. A.

[13] Biccai S., Barwich S., Boland D., Harvey A., Hanlon D., McEvoy N., Coleman J.N. (2018). Exfoliation of 2D materials by high shear mixing. 2D Mater..

[14] Yuan H., Liu X., Ma L., Gong P., Yang Z., Wang H., Wang J., Yang S. (2016). High efficiency shear exfoliation for producing high-quality, few-layered MoS2 nanosheets in a green ethanol/water system. RSC Adv..

[15] Shen J., He Y., Wu J., Gao C., Keyshar K., Zhang X., Yang Y., Ye M., Vajtai R., Lou J. (2015). Liquid Phase Exfoliation of Two-Dimensional Materials by Directly Probing and Matching Surface Tension Components. Nano Lett..

[16] Gai Y., Wang W., Xiao D., Tan H., Lin M., Zhao Y. (2018). Exfoliation of Graphite into Graphene by a Rotor–Stator in Supercritical CO2: Experiment and Simulation. Ind. Eng. Chem. Res..

[17] Kaushik V., Wu S., Jang H., Kang J., Kim K., Suk J.W. (2018). Scalable Exfoliation of Bulk MoS2 to Single- and Few-Layers Using Toroidal Taylor Vortices. Nanomaterials.

[18] Hall S., Cooke M., Pacek A.W., Kowalski A.J., Rothman D. (2011). Scaling up of silverson rotor–stator mixers. Can. J. Chem. Eng..

[19] Ovchinnikov D., Allain A., Huang Y.-S., Dumcenco D., Kis A. (2014). Electrical Transport Properties of Single-Layer WS2. ACS Nano.

[20] Pawbake A.S., Waykar R.G., Late D.J., Jadkar S.R. (2016). Highly transparent wafer-scale synthesis of crystalline WS2 nanoparticle thin film for photodetector and humidity-sensing applications. ACS App. Mater. Inter..

[21] Zhang C., Tan J., Pan Y., Cai X., Zou X., Cheng H.-M., Liu B. (2019). Mass production of 2D materials by intermediate-assisted grinding exfoliation. Nat. Sci. Rev..

[22] Tubon Usca G., Vacacela Gomez C., Guevara M., Tene T., Hernandez J., Molina R., Tavolaro A., Miriello D., Caputi L.S. (2019). Zeolite-Assisted Shear Exfoliation of Graphite into Few-Layer Graphene. Crystals.

[23] Kameda T., Ohkuma K., Oka S. (2019). Polytetrafluoroethylene (PTFE): A resin material for possible use in dental prostheses and devices. Dent. Mater. J..

[24] Xu D., Xu P., Zhu Y., Peng W., Li Y., Zhang G., Zhang F., Mallouk T.E., Fan X. (2018). High Yield Exfoliation of WS2 Crystals into 1–2 Layer Semiconducting Nanosheets and Efficient Photocatalytic Hydrogen Evolution from WS2/CdS Nanorod Composites. ACS App. Mater. Inter..

[25] Adilbekova B., Lin Y., Yengel E., Faber H., Harrison G., Firdaus Y., El-Labban A., Anjum D.H., Tung V., Anthopoulos T.D. (2020). Liquid phase exfoliation of MoS2 and WS2 in aqueous ammonia and their application in highly efficient organic solar cells. J. Mater. Chem. C.

[26] Han C., Zhang Y., Gao P., Chen S., Liu X., Mi Y., Zhang J., Ma Y., Jiang W., Chang J. (2017). High-Yield Production of MoS2 and WS2 Quantum Sheets from Their Bulk Materials. Nano Lett..

[27] Ma L., Liu Z., Cheng Z.-L. (2020). Scalable exfoliation and friction performance of few-layered WS2 nanosheets by microwave-assisted liquid-phase sonication. Ceram. Int..

[28] Pagona G., Bittencourt C., Arenal R., Tagmatarchis N. (2015). Exfoliated semiconducting pure 2H-MoS2 and 2H-WS2 assisted by chlorosulfonic acid. Chem. Commun..

[29] Coleman J.N., Lotya M., O’Neill A., Bergin S.D., King P.J., Khan U., Young K., Gaucher A., De S., Smith R.J. (2011). Two-Dimensional Nanosheets Produced by Liquid Exfoliation of Layered Materials. Science.

[30] Devos O., Mouton N., Sliwa M., Ruckebusch C. (2011). Baseline correction methods to deal with artifacts in femtosecond transient absorption spectroscopy. Anal. Chim. Acta.

[31] Lieber C.A., Mahadevan-Jansen A. (2003). Automated Method for Subtraction of Fluorescence from Biological Raman Spectra. Appl. Spectrosc..

[32] Jung J.H., Park C.-H., Ihm J. (2018). A Rigorous Method of Calculating Exfoliation Energies from First Principles. Nano Lett..

[33] Thripuranthaka M., Kashid R.V., Sekhar Rout C., Late D.J. (2014). Temperature dependent Raman spectroscopy of chemically derived few layer MoS2 and WS2 nanosheets. App. Phys. Lett..

[34] Schmälzlin E., Moralejo B., Rutowska M., Monreal-Ibero A., Sandin C., Tarcea N., Popp J., Roth M.M. (2014). Raman imaging with a fiber-coupled multichannel spectrograph. Sensors.

[35] Koenig J.L., Boerio F.J. (1969). Raman Scattering and Band Assignments in Polytetrafluoroethylene. J. Chem. Phys..

[36] Zhang Y., Xu L., Walker W.R., Tittle C.M., Backhouse C.J., Pope M.A. (2017). Langmuir films and uniform, large area, transparent coatings of chemically exfoliated MoS2 single layers. J. Mater. Chem. C.

[37] Berkdemir A., Gutiérrez H.R., Botello-Méndez A.R., Perea-López N., Elías A.L., Chia C.-I., Wang B., Crespi V.H., López-Urías F., Charlier J.-C. (2013). Identification of individual and few layers of WS2 using Raman Spectroscopy. Sci. Rep..

