# Supplementary material for: Additive-Enhanced Exfoliation for High-Yield 2D Materials Production
Source: Nanomaterials (Basel). 2021 Feb 28;11(3):601. doi: 10.3390/nano11030601 (PMC7997357; doi:10.3390/nano11030601)
Supplement: Supplementary file 1 [file nanomaterials-11-00601-s001.pdf]

nanomaterials

MDPI

Supplementary Materials

Additive-Enhanced Exfoliation for High-Yield 2D Materials Production

Dinh-Tuan Nguyen [1], Hsiang-An Ting [2], Yen-Hsun Su [1], Mario Hofmann [3,*] and Ya-Ping Hsieh [4,*]

[1] Department of Materials Science and Engineering, National Cheng Kung University, Tainan 70101, Taiwan; tuanngdvn@gmail.com (D.-T.N.); yhsu@mail.ncku.edu.tw (Y.-H.S.)
[2] Department of Mechanical Engineering, National Chiao Tung University, Hsinchu 30010, Taiwan; andyting86@gmail.com
[3] Department of Physics, National Taiwan University, Taipei 10617, Taiwan
[4] Institute of Atomic and Molecular Sciences, Academia Sinica, Taipei 10617, Taiwan
* Correspondence: mario@phys.ntu.edu.tw (M.H.); yphsieh@gate.sinica.edu.tw (Y.-P.H.)

1. Comparison of Additives

Table S1. Comparison of various additives.

Criteria	Nickel	Silica	D.E.	Zeolite	PTFE
Solvent stability	+	o	o	+	+
Mechanical stability	o	–	–	–	+
Removability	+	O	o	–	o
Size	–	+	o	+	+

2. Simulation of Shear Enhancement in AAE

Finite element simulation was conducted to model the velocity distribution in a reactor under rotation conditions. For this, the motion of a rectangular rotor in proximity to a stationary wall was simulated and the AAE condition was emulated by the introduction of a small amount of moving spherical particles. The local shear rate was then extracted as the spatial derivative of the fluid velocity. The increase in shear rate for AAE compared to a conventional reactor can be observed in Figure 1c, which corroborates the observed enhancement in exfoliation yield for AAE. Furthermore, we modified the rotation speed and demonstrated that the shear rate for AAE is consistently larger than for conventional conditions.

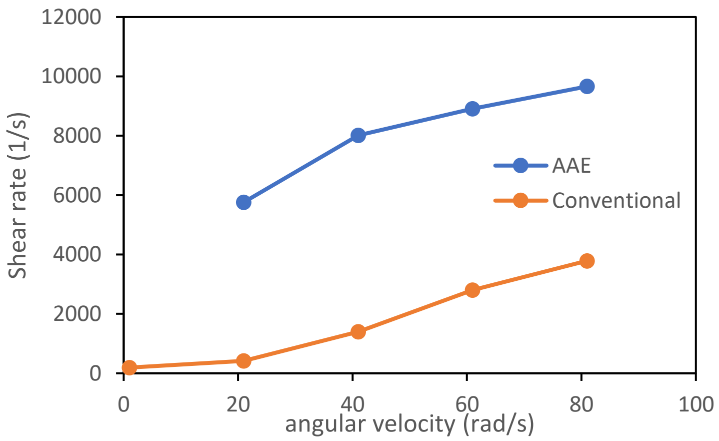

Figure S1. Calculated shear rates of AAE (blue line) and conventional exfoliation (orange line).

3. Weight Measurement and Yield Comparison

As exfoliation produces nanosheets with a wide thickness distribution, to compare the yield between methods, a threshold must be set for what count as 'exfoliated' material. In this instance, we consider the material not sedimented after 2 h of 1200 krpm centrifugation as exfoliated. Another problem is that the process of removing PTFE via vacuum filtration also removes some WS_2. Moreover, NMP is not easily evaporated. Therefore, we measured the unexfoliated material instead by weighing materials in the solution in several steps, which include the material left at the rotor head w_{sed} and stator cage w_{stator} of the mixer, the sediment in the centrifuge tube $w_{centrifuge}$ and the material retained by the filter membrane $w_{filtered}$. The exfoliated material is then equaled to the initial total weight minus the unexfoliated part (along with additives, in the case of AAE):

$$w_{exfoliated} = w_{initial} - \left(w_{sed} + w_{rotor} + w_{stator} + w_{centrifuge} + w_{filtered}\right) - w_{additive} \quad (1)$$

For AAE, this weight amounts to ~2.81g exfoliated material produced from 9g initial material, corresponding to ~31% yield.

4. WS_2 Flake Size Distribution

By particle analysis of the AFM images of the WS_2 sample, we obtained the flake size and thickness distribution.

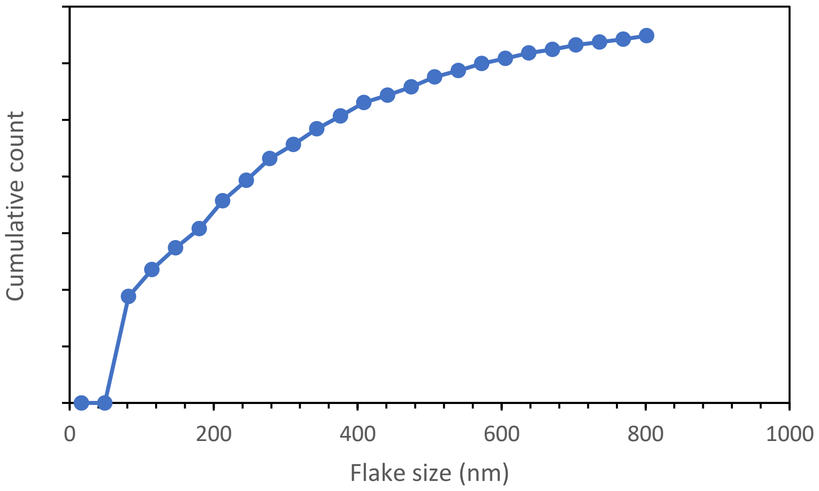

Figure S2. WS_2 flake size distribution.

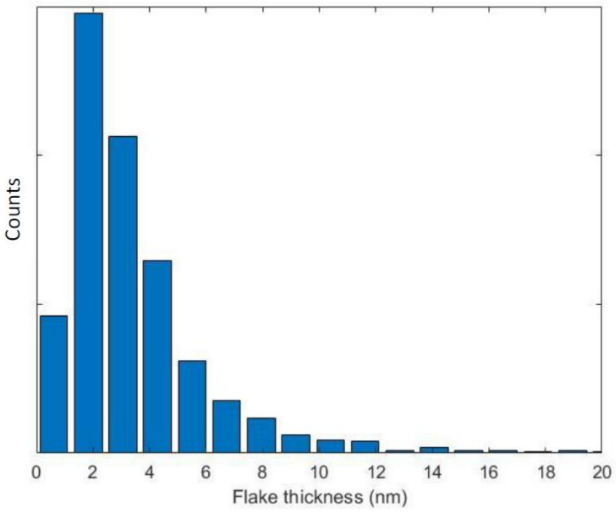

Figure S3. WS_2 thickness distribution.
